# Loss of the AE3 Cl^−^/HCO^−^_3_ exchanger in mice affects rate-dependent inotropy and stress-related AKT signaling in heart

**DOI:** 10.3389/fphys.2013.00399

**Published:** 2013-12-31

**Authors:** Vikram Prasad, John N. Lorenz, Valerie M. Lasko, Michelle L. Nieman, Nabeel J. Al Moamen, Gary E. Shull

**Affiliations:** ^1^Departments of Molecular Genetics, Biochemistry and Microbiology, University of Cincinnati College of MedicineCincinnati, OH, USA; ^2^Departments of Cellular and Molecular Physiology, University of Cincinnati College of MedicineCincinnati, OH, USA; ^3^Genetic Laboratory, Department of Pathology, Salmaniya Medical ComplexManama, Bahrain

**Keywords:** *Slc4a3*, *Slc4a4*, NBCe1, SERCA2, NCX1, costamere, protein kinase B

## Abstract

Cl^−^/HCO^−^_3_ exchangers are expressed abundantly in cardiac muscle, suggesting that HCO^−^_3_ extrusion serves an important function in heart. Mice lacking Anion Exchanger Isoform 3 (AE3), a major cardiac Cl^−^/HCO^−^_3_ exchanger, appear healthy, but loss of AE3 causes decompensation in a hypertrophic cardiomyopathy (HCM) model. Using intra-ventricular pressure analysis, *in vivo* pacing, and molecular studies we identified physiological and biochemical changes caused by loss of AE3 that may contribute to decompensation in HCM. AE3-null mice had normal cardiac contractility under basal conditions and after β-adrenergic stimulation, but pacing of hearts revealed that frequency-dependent inotropy was blunted, suggesting that AE3-mediated HCO^−^_3_ extrusion is required for a robust force-frequency response (FFR) during acute biomechanical stress *in vivo*. Modest changes in expression of proteins that affect Ca^2+^-handling were observed, but Ca^2+^-transient analysis of AE3-null myocytes showed normal twitch-amplitude and Ca^2+^-clearance. Phosphorylation and expression of several proteins implicated in HCM and FFR, including phospholamban (PLN), myosin binding protein C, and troponin I were not altered in hearts of paced AE3-null mice; however, phosphorylation of Akt, which plays a central role in mechanosensory signaling, was significantly higher in paced AE3-null hearts than in wild-type controls and phosphorylation of AMPK, which is affected by Akt and is involved in energy metabolism and some cases of HCM, was reduced. These data show loss of AE3 leads to impaired rate-dependent inotropy, appears to affect mechanical stress-responsive signaling, and reduces activation of AMPK, which may contribute to decompensation in heart failure.

## Introduction

Despite the abundance of Cl^−^/HCO^−^_3_ exchangers in heart, which include the AE1, AE2, and AE3 anion exchangers (*Slc4a1–3*) and putative anion transporter 1 (PAT1; *Slc26a6*) (Kudrycki et al., 1990; Knauf et al., 2001; Wang et al., 2002; Alvarez et al., 2004), their role in regulating cardiac function remains poorly understood. In epithelial tissues, Cl^−^/HCO^−^_3_ exchangers, functioning coordinately with Na^+^-loading mechanisms, facilitate pH-neutral Na^+^ and Cl^−^ ion fluxes (Melvin et al., 2005; Alper, 2009; Gawenis et al., 2010), thereby contributing to anion secretion and volume regulation. In erythrocytes on the other hand, AE1 plays a key structural role in maintaining the cytoskeletal stability of erythrocyte membranes (Peters et al., 1996; Southgate et al., 1996) in addition to being involved in gas exchange (Bruce et al., 2003; Jensen, 2004). Anion Exchanger Isoform 3 (AE3), which has a cardiac-specific variant (Linn et al., 1992) and is most highly expressed in heart (Kudrycki et al., 1990), has been localized to both t-tubules and sarcolemma, with apparent foci of expression at costameres (Alvarez et al., 2007a), which are complexes involved in mechanical stress sensing and mechanotransduction (Samarel, 2005). However, an essential role for AE3 in heart is not self-evident as AE3-null mice appear healthy and exhibit normal cardiac performance under basal conditions and after β-adrenergic stimulation (Prasad et al., 2008).

The first direct evidence that AE3 plays an important role in heart was the finding that the additional loss of AE3 in NKCC1 Na^+^-K^+^-2Cl^−^ cotransporter-null mice led to impaired contractility (Meyer et al., 2002; Prasad et al., 2008). More recently, loss of AE3 was shown to dramatically increase the rate of decompensation in a transgenic mouse α-tropomyosin Glu180Gly (TM180) hypertrophic cardiomyopathy (HCM) model. Although the degree of hypertrophy and expression of markers of hypertrophy were not increased, loss of AE3 led to reduced contractility and relaxation and a sharply reduced life-span (Al Moamen et al., 2011). These findings showed that loss of AE3 expression and activity, although benign under normal circumstances, leads to changes that contribute to decompensation in HCM and eventual heart failure.

To identify changes that might contribute to the predisposition to heart failure we analyzed AE3-null mice on an FVB/N background, which was used in our previous studies of the interactions of AE3 ablation and TM180-induced HCM (Al Moamen et al., 2011). Cardiovascular performance of AE3-null mice on the FVB/N background was normal under both basal conditions and in response to β-adrenergic stimulation, as seen previously in mice on a mixed 129Svj and Black Swiss background (Prasad et al., 2008). However, when heart rates were increased by pacing *in vivo*, a procedure that subjects the heart to acute biomechanical stress, the positive force-frequency relationship/response (FFR) was blunted, a common occurrence in heart failure (Rossman et al., 2004). To better understand the role of AE3 in heart disease and rate-dependent inotropy, we also analyzed, under both basal conditions and after *in vivo* pacing at elevated heart rates, a number of phosphorylated proteins that have been implicated in heart disease and that function under conditions of varying biomechanical stress. Consistent with an altered response to biomechanical stress, phosphorylation of Akt was increased in AE3-null hearts when subjected to elevated heart rates but not at basal heart rates. In contrast, phosphorylation of AMPK, which serves as a master regulator of energy metabolism (Zaha and Young, 2012) and is affected by Akt activation, was reduced during pacing. These results show that AE3 ablation impacts inotropic responses and signaling mechanisms elicited by acute biomechanical stress *in vivo* and suggest that AE3-mediated Cl^−^/HCO^−^_3_ exchange plays an important protective role in conditions leading to HCM.

## Materials and methods

### Generation of mutant mice

Generation and genotype analysis of AE3-null mice has been described previously (Prasad et al., 2008). Mice used in this study were on an inbred FVB/N background. All procedures conformed to guidelines published by the National Institutes of Health (*Guide for the Care and Use of Laboratory Animals*; Publication No. 86-23, revised 1996) and were approved by the Institutional Animal Care and Use Committee at the University of Cincinnati.

### Analysis of *in vivo* cardiovascular performance and force-frequency relationships

Analysis of cardiovascular function was performed as described previously (Lorenz and Robbins, 1997; Periasamy et al., 1999). Intraventricular pressure (IVP) measurements in the left ventricle of mice anesthetized with ketamine and inactin (50 and 100 μg/g bodyweight) were carried out using a Millar high-fidelity pressure transducer inserted via the right carotid artery. The right femoral artery was cannulated with a fluid-filled catheter connected to a pressure transducer for blood pressure measurements. Drugs were infused via the right femoral vein. Anesthetized mice were subjected to closed-chest atrial pacing as described previously (D'Angelo et al., 1997; Al Moamen et al., 2011). Heart rates were electrically paced, with increments of 50 beats per minute (bpm), and cardiovascular parameters were recorded as described above.

### Real-time PCR analysis

Total RNA was isolated from wild-type and AE3-null hearts as previously described (Al Moamen et al., 2011) and cDNA generated using oligo dT primers and the Superscript III First-strand system (Invitrogen; 18080-051). Real-time PCR (RT-PCR) analysis of cDNA was carried out using the Absolute Blue qPCR SYBR mix (Thermo Scientific; AB4219/B), the Opticon2 DNA Engine (MJ Research Inc.), and QuantiTect primer assays (Qiagen) targeted against mRNA for the following genes: *Slc4a4* encoding NBCe1 (Primer assay: QT01053675), *Slc4a7* encoding NBCn1 (Primer assay: QT01037036), and *Slc26a6* encoding PAT1 (Primer assay: QT01661828). Expression of individual genes was normalized to expression levels of *Gapdh*, which were determined as previously described (Al Moamen et al., 2011). Results were analyzed using the Opticon Monitor Analysis Software (Ver. 3.1).

### Analysis of Ca^2+^ transients

Ventricular myocytes were isolated from adult mouse hearts as described previously (O'Connell et al., 2003) using an enzyme mixture of Liberase Blendzyme IV (Roche) at 0.25 mg/mL and trypsin (Invitrogen) at 0.14 mg/mL. Cells were loaded with Fura2-AM (Molecular Probes Inc.; Eugene, OR) at a final concentration of 2 μ M for 15–20 min at room temperature and fluorescence measurements were made using a dual-beam spectrofluorophotometer (PTI International, Birmingham, NJ) after field stimulation at 0.5 Hz (Ji et al., 2006). Data were acquired using Felix 3.01 acquisition software (PTI International) and tracings of Ca^2+^ transients were obtained by calculating fluorescence ratios (340:380 nm). Ionoptix Ionwizard Analysis Software (IonOptix LLC, Milton, MA) was used for analysis.

### Immunoblot analysis

Hearts were harvested from anesthetized mice (15 μl of 2.5% Avertin/g bodyweight) and total homogenates prepared as described previously (Prasad et al., 2008). For analyses of phosphorylated proteins in instrumented control and paced hearts (550 bpm), left ventricular tissue was collected from mice that were surgically instrumented as described above. Hearts were paced from baseline to 550 bpm, with increments of 50 bpm. After recording of parameters, the chest-cavity was opened as pacing at 550 bpm was continued, and ventricular tissue at the apex of the heart was rapidly excised and immediately frozen in liquid N_2_ prior to preparation of total heart homogenates. Protein concentrations were estimated using a modified Bradford assay (Thermo Scientific). Proteins were resolved on reducing SDS polyacrylamide gels, transferred to nitrocellulose or PVDF membranes and incubated with specific primary antibodies followed by corresponding secondary antibodies (KPL, Gaithersburg MD, USA). Protein bands were visualized using the KPL LumiGlo chemiluminescent substrate system (KPL, Gaithersburg MD, USA). The primary antibodies for SERCA2a, phospholamban (PLN), phospho-Ser16 and phospho-Thr17 of PLN, the NCX1 Na^+^/Ca^2+^ exchanger, the L-type Ca^2+^ channel α2 subunit, the cardiac ryanodine receptor (RyR2), and sarcomeric actin (as a loading control) were the same as used previously (Prasad et al., 2008; Al Moamen et al., 2011). Additional antibodies used were those for the NHE1 Na^+^/H^+^ exchanger (MAB3140, clone 4E9: Millipore); MyBP-C (sc-67353: Santa Cruz Biotechnology); phospho-Ser282 myosin binding protein-C (MyBP-C) (ALX-215, Alexis Biochemicals); total and phospho-Ser22/23 cardiac troponin I (TnI) (4002 and 4004, respectively, Cell Signaling Technology); total and phospho-Ser473 Akt (9272 and 9271, respectively, Cell Signaling Technology); total and phospho-Thr172 AMPK (2532 and 2535, respectively, Cell Signaling Technology; and NBCe1 (11867, Cell Signaling).

### Statistics

Values are presented as means ± standard error (SE). For group comparisons, a mixed factor analysis of variance with repeated measures on the second factor was used. Individual comparisons were performed using two-tailed Student's *t*-test, and a *p*-value of <0.05 was considered significant.

## Results

### Cardiovascular performance is normal in AE3-null mice on the FVB/N background

**Figure 1:**
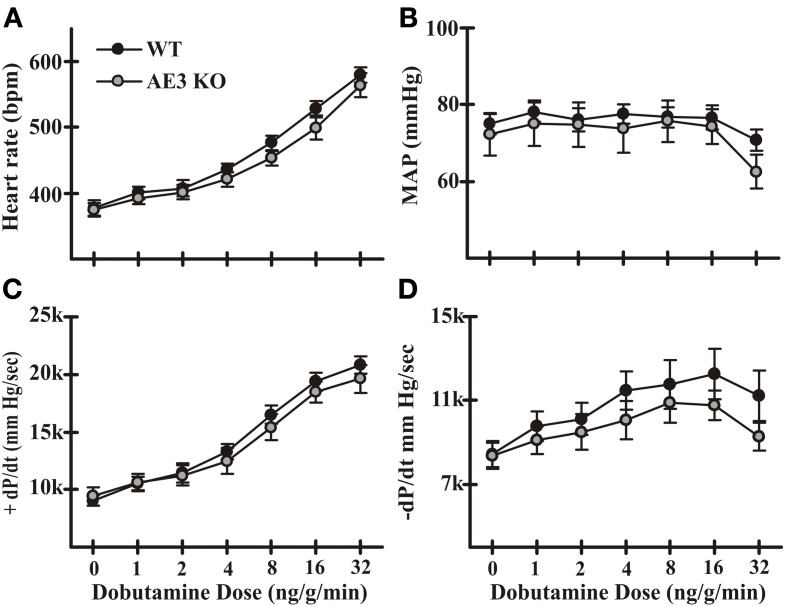
**Cardiovascular performance in wild-type and AE3-null mice**. Intraventricular and arterial pressures were measured in instrumented anesthetized mice using transducers inserted in the left ventricle and right femoral artery, respectively. Results shown include: **(A)** heart rate; **(B)** mean arterial pressure (MAP); **(C)** +dP/dt; and **(D)** −dP/dt under both basal conditions and upon β-adrenergic stimulation by infusion of dobutamine via the femoral vein. Values shown are means ± SE, *n* = 11 for WT, 9 for AE3-null (KO); no significant differences were observed between WT and KO mice.

In a previous study we analyzed the effects of the AE3 null mutation using mice of a mixed 129Svj and Black Swiss background and found no significant differences in cardiovascular performance (Prasad et al., 2008). However, contractile differences have been shown to exist between mice of different genetic backgrounds (Kadambi et al., 1999). Of particular relevance, the inotropic effects of β-adrenergic stimulation are significantly different between wild-type (WT) mice of the 129Svj and Black Swiss mixed background and the inbred FVB/N background, with the mixed background mice achieving much greater values for +dP/dt in response to dobutamine. Therefore, we performed IVP analysis of WT and AE3-null mice on the inbred FVB/N background that was used to analyze the effects of AE3 ablation in the TM180 HCM model (Al Moamen et al., 2011). Although mean absolute values of −dP/dt for AE3-null mice were somewhat lower than in WT mice, the differences were not significant and no significant differences in other cardiovascular parameters, including heart rate, mean arterial pressure, and +dP/dt, were observed between WT and AE3-null mice under either basal conditions or after β-adrenergic stimulation (Figure 1).

### Positive force-frequency relationship is blunted in AE3-null mice

A common feature of heart failure is a reduction in the positive FFR (Rossman et al., 2004). In our studies of the effects of AE3 ablation in the TM180 HCM model (Al Moamen et al., 2011), FFR and the ability to pace hearts to higher frequencies appeared to be more affected in double mutants, although this could have been a function of the more severe heart failure. To assess FFR in WT and AE3-null mice, animals were surgically instrumented and cardiovascular parameters were recorded as hearts were paced *in vivo* from baseline to 550 bpm. In an initial set of mice (*n* = 7 pairs) in which pacing up to 750 bpm was attempted, AE3-null mice tended to be less able than WT mice to sustain higher heart rates. Thus, in subsequent experiments, pacing was limited to 550 bpm, and heart samples were collected for biochemical studies described in later sections.

**Figure 2:**
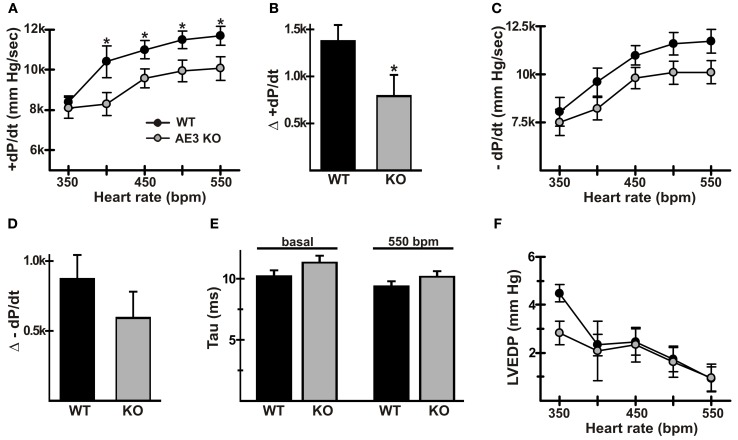
**Force-frequency relationships in wild-type and AE3-null mice**. Pressure measurements were recorded in anesthetized WT and AE3-null (KO) mice that were instrumented as described in Figure 1, and subjected to closed-chest atrial pacing from 350 to 550 bpm. Results show: **(A)** changes in +dP/dt upon pacing; **(B)** changes in +dP/dt represented as Δ +dP/dt_max_ and calculated as the difference between values at 550 bpm and baseline heart rate, which was comparable between genotypes (WT: 403 ± 10 bpm; AE3-null: 396 ± 9 bpm, *p* = 0.6); **(C)** changes in −dP/dt upon pacing; **(D)** changes in −dP/dt represented as Δ −dP/dt and calculated as the difference between values at 550 bpm and baseline heart rate; **(E)** Tau (relaxation time constant) values under basal and paced conditions; **(F)** changes in left ventricular end diastolic pressure (LVEDP) upon pacing. Values shown are means ± SE, *n* = 19 mice for each genotype except for dP/dt values at 350 bpm (*n* = 4 for each genotype) and 400 bpm (*n* = 9 for each genotype). ^*^p < 0.05.

Pacing in WT mice led to an increase in +dP/dt, from 8402 ± 296 mm Hg/s at 350 bpm to 11700 ± 470 mm Hg/s at 550 bpm; the corresponding increase in mutant mice was from 8112 ± 534 at 350 bpm to 10066 ± 588 mm Hg/s at 550 bpm (Figure 2A). Because baseline heart rate for some mice was above 350 or 400 bpm, the significant blunting of rate-dependent inotropy was also quantified as the difference between +dP/dt at baseline and 550 bpm (Δ +dP/dt). Δ +dP/dt was 1434 ± 210 mm Hg/s in WT mice, whereas in AE3-null mice it was 696 ± 297 mm Hg/s (Figure 2B). Basal heart rates were comparable between WT and AE3-null hearts (WT: 403 ± 10 bpm; AE3-null: 396 ± 9 bpm, *p* = 0.6).

Blood pressure and left ventricular systolic pressure in FVB/N mice remain at a relatively constant level as heart rate increases (for example, see mean arterial pressure and corresponding heart rates in Figure 1) and do not exhibit a significant frequency response. Consistent with this observation, mean arterial pressures (in mm Hg ± SD) changed very little between baseline (WT, 79.0 ± 8.1; AE3-null, 72.1 ± 10.2) and 550 bpm (WT, 84.6 ± 8.1; AE3-null 75.7 ± 11.2). Similarly, left ventricular systolic pressure exhibited essentially no change in either genotype between baseline (WT, 96.5 ± 11.2; AE3-null, 88.3 ± 11.9) and 550 bpm (WT, 99.1 ± 10.6; AE3-null, 88.8 ± 12.9).

In some forms of heart failure, a reduction in frequency-dependent acceleration of relaxation (FDAR), indicative of impaired relaxation, is observed (Wachter et al., 2009). Although pacing-induced changes in −dP/dt were blunted in AE3-null mice (Figure 2C) and the Δ −dP/dt at 550 bpm—baseline (Figure 2D) was reduced in AE3-null mice (−870 ± 172 and −592 ± 188 mm Hg/s in WT and AE3-null, respectively), the differences were not significant. Also, tau values (Figure 2E), a more reliable measure of relaxation (Weiss et al., 1976), were not significantly different under basal conditions (10.2 ± 0.5 and 11.3 ± 0.6 ms in WT and AE3-null, respectively) or when paced at 550 bpm (9.4 ± 0.4 and 10.2 ± 0.5 ms in WT and AE3-null, respectively). Finally, left ventricular end diastolic pressure during pacing was similar in WT and AE3 mutant mice (Figure 2F).

### Expression of proteins that affect Ca^2+^-handling is altered in AE3-null mice

**Figure 3:**
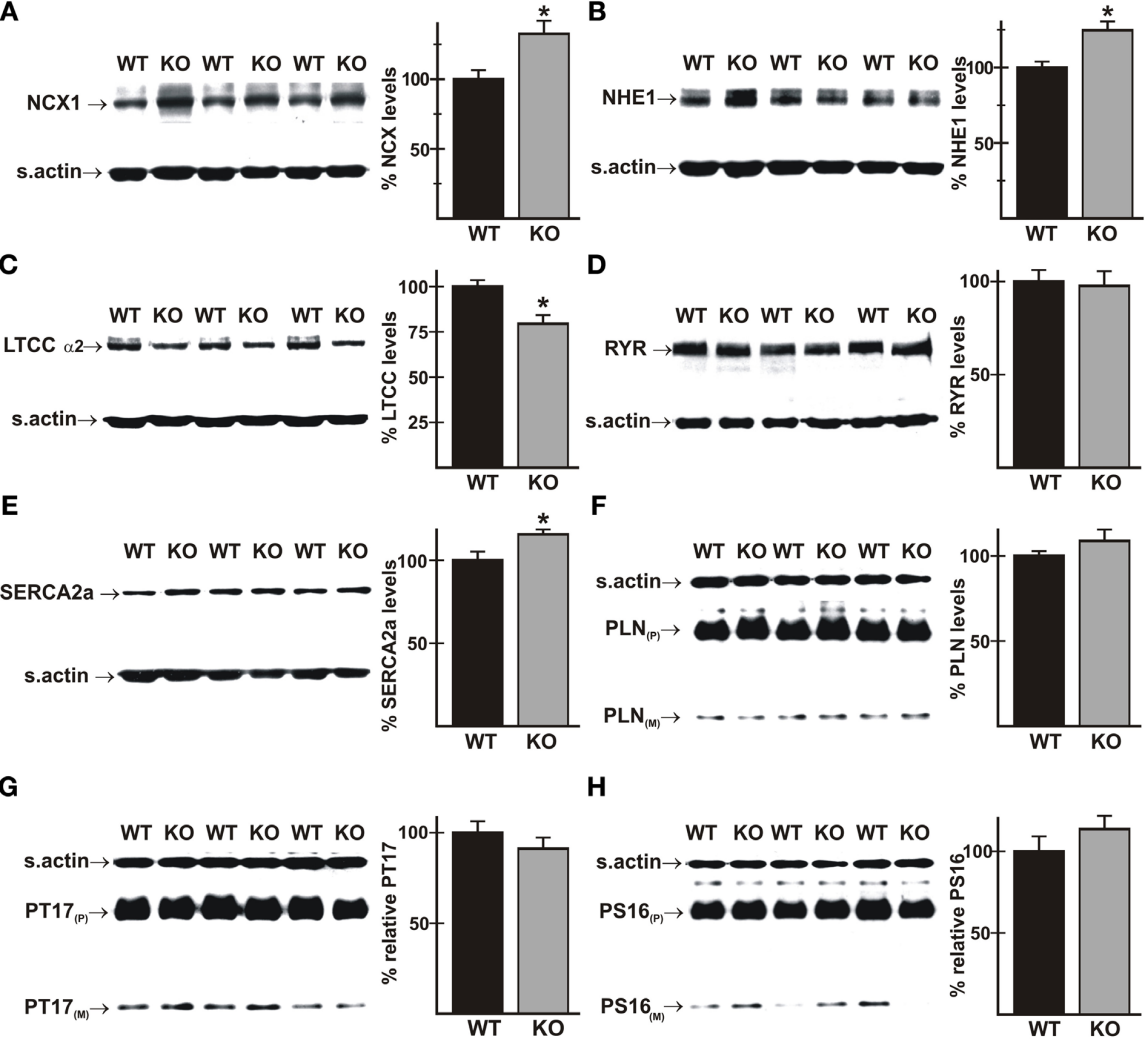
**Expression of proteins that affect Ca^2+^-handling in wild-type and AE3-null hearts**. Proteins from total homogenates of WT and AE3-null (KO) hearts were subjected to immunoblot and densitometric analyses; levels of each protein were normalized to levels of sarcomeric actin (s.actin) in the same sample. Results show representative immunoblots, with sarcomeric actin controls, and percent expression levels of: **(A)** NCX1 Na^+^/Ca^2+^ exchanger; **(B)** NHE1 Na^+^/H^+^ exchanger; **(C)** L-Type Ca^2+^ channel α2 subunit; **(D)** ryanodine receptor (RYR); **(E)** SERCA2a Ca^2+^ pump; **(F)** phospholamban (PLN); **(G)** PLN phosphorylated on Thr17 (PT17); and **(H)** PLN phosphorylated on Ser16 (PS16). P and M indicate pentameric and monomeric forms of PLN, respectively. Values shown are mean ± SE, *n* = at least 4 mice for each genotype. ^*^p < 0.05 vs. WT.

Perturbations in Ca^2+^ handling occur frequently in heart disease, and could therefore be a contributing factor in an underlying predisposition to heart failure. Immunoblot analyses using total cardiac homogenates revealed modest changes in proteins known to affect Ca^2+^-handling. Mutant hearts exhibited increased levels of the NCX1 Na^+^/Ca^2+^ exchanger (Figure 3A) and the NHE1 Na^+^/H^+^ exchanger (Figure 3B), which is increased in cardiac hypertrophy and can affect Ca^2+^ handling via NCX1-mediated Na^+^-loading (Karmazyn et al., 2008). Expression of the α2 subunit of the L-type Ca^2+^ channel (LTCC) was reduced (Figure 3C), and cardiac ryanodine receptor (RYR2) levels were unaltered (Figure 3D). There was a small but significant increase in SERCA2 in mutant hearts (Figure 3E; 116 ± 3% of WT levels), which might be expected to improve Ca^2+^ handling, but levels of total phospholamban (PLN) and PLN phosphorylated on either Thr17 or Ser16 (Figures 3F–H) were unaltered.

### NBCe1 mRNA and protein expression is down-regulated in AE3-null mice

**Figure 4:**
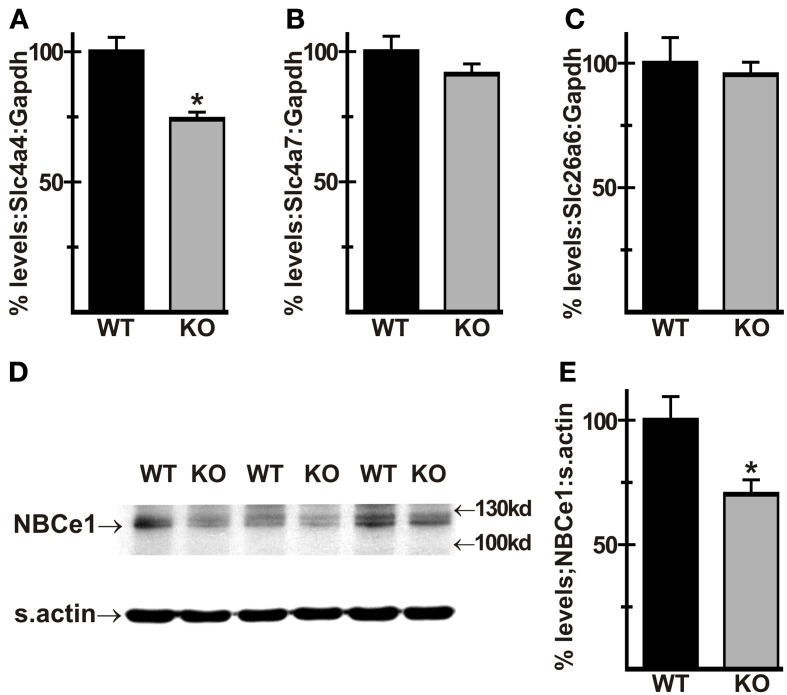
**Expression of HCO^−^_3_ transporters in AE3-null hearts**. RT-PCR analysis of cDNA generated from total RNA of WT and AE3-null (KO) hearts was carried out to determine mRNA levels the *Slc4a4* and *Slc4a7* Na^+^/HCO^−^_3_ cotransporters and *Slc26a6*, encoding the anion exchanger PAT1. When normalized to Gapdh levels, expression of *Slc4a4* was downregulated in AE3-null hearts **(A)**, while *Slc4a7* **(B)** and *Sl26a6* **(C)** levels showed no change. Total cardiac homogenates of WT and KO hearts were subjected to immunoblot and densitometric analyses as described in Figure 3; results show that NBCe1 protein expression, when normalized to levels of sarcomeric actin (s.actin) was reduced in AE3-null hearts **(D,E)**. Values shown are mean ± S.E. *n* = at least 10 mice of each genotype for RT-PCR analysis and at least 7 mice of each genotype for immunoblot analysis. ^*^p < 0.02 vs. WT.

To address the possibility that loss of AE3 altered expression of other major HCO^−^_3_ transporters in heart, RT-PCR analysis of total RNA from WT and AE3-null hearts was carried out. Results showed that mRNA levels of *Slc4a4*, which encodes the electrogenic NBCe1 Na^+^/HCO^−^_3_ cotransporter were downregulated in AE3 null hearts (74 ± 3% of WT levels; Figure 4A). In contrast, no change was observed in expression levels of *Slc4a7*, which encodes the electroneutral NBCn1 Na^+^/HCO^−^_3_ cotransporter, and *Slc26a6*, which codes for PAT1 (Figures 4B, C). Consistent with these results, immunoblot analysis of total cardiac homogenates revealed that NBCe1 protein levels, when normalized to sarcomeric actin, were also reduced in AE3-null hearts (70 ± 6% of WT levels; Figures 4D, E).

### Ca^2+^ transients are normal in AE3-null myocytes

**Figure 5:**
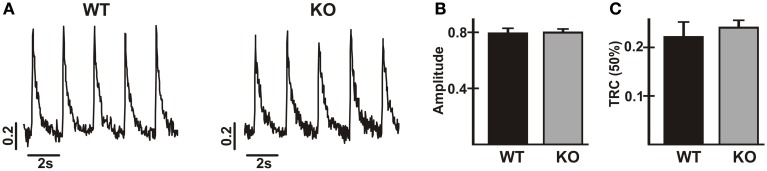
**Analysis of Ca^2+^-transients in cardiac myocytes from wild-type and AE3-null mice**. Ca^2+^-transients were calculated as 340/380 ratios from fluorescence measurements in isolated myocytes from WT and AE3-null (KO) mice loaded with Fura-2. Results show: **(A)** representative tracings; **(B)** Ca^2+^-transient amplitudes; and **(C)** time to 50% recovery of Ca^2+^-transients in response to electrical field-stimulation at 0.5 Hz. Values are means ± SE, *n* = 3 mice per genotype (at least 36 cells/genotype).

In previous studies, Ca^2+^ transients were reduced and decay times were prolonged in isolated ventricular myocytes from TM180 HCM mice carrying the AE3-null mutation (Al Moamen et al., 2011), although it seemed likely that this was due to the greater severity of the disease state in AE3/TM180 double mutant mice. To assess the effects of AE3 ablation alone on Ca^2+^ handling, Ca^2+^-transients in response to field-stimulation at 0.5 Hz were analyzed in isolated myocytes of WT and AE3-null hearts (Figure 5A). Neither the amplitude of the Ca^2+^-transient (Figure 5B) nor the time to 50% decay (Figure 5C) was altered, indicating that Ca^2+^ release and sequestration were relatively normal in AE3-null myocytes. However, it should be noted that during stimulation the AE3-null myocytes exhibited a much greater tendency to lose their characteristic shape and round up (see Discussion).

### Phosphorylation of PLN Thr17 is not altered in AE3-null hearts during pacing

**Figure 6:**
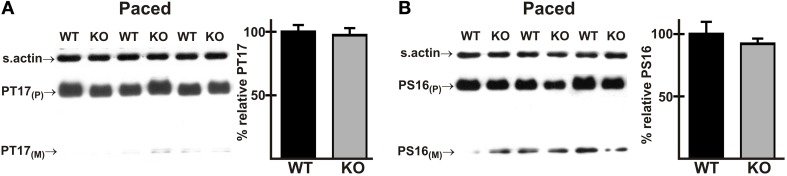
**Phosphorylation of phospholamban in wild-type and AE3-null hearts subjected to atrial pacing *in vivo***. WT and AE3-null (KO) mice were anesthetized, instrumented, and subjected to *in vivo* pacing as described in Figure 2. Ventricular tissue was harvested during pacing at 550 bpm, and immunoblot and densitometric analyses of homogenates were carried out to determine levels of **(A)** PLN phosphorylated on Thr17 (PT17) and **(B)** PLN phosphorylated on Ser16 (PS16). Results show representative immunoblot and percent relative levels of phosphorylated PLN (normalized to total PLN levels). Note that PLN phosphorylation did not differ between the two genotypes under basal conditions (Figures 3G, H). Values shown are means ± SE, *n* = 8 for each genotype.

A rare mutation in PLN has been implicated in HCM (Landstrom et al., 2011), and phosphorylation of Thr17 and Ser16 of PLN has been shown to be reduced in a dog model of HCM (Mishra et al., 2003). Furthermore, stimulation of Thr17 phosphorylation is required for a robust FFR in isolated mouse cardiomyocytes (Zhao et al., 2004), albeit at much lower frequencies than those occurring *in vivo*. Immunoblotting showed that phosphorylation of Thr17 was not significantly different in WT and AE3-null hearts under basal conditions (Figure 3G) or after *in vivo* pacing at 550 bpm (Figure 6A). Similar studies of phosphorylation of Ser16 of PLN, which did not differ between WT and AE3-null mice under basal conditions (Figure 3H), revealed no differences between the two genotypes during pacing at 550 bpm (Figure 6B). These results and the modest increase in levels of SERCA2 (Figure 3E), which is often reduced in heart failure (Kho et al., 2012), argue against a primary role for an *in vivo* deficit in sarcoplasmic reticulum Ca^2+^ handling in the predisposition of AE3-null mice to heart failure in the HCM model.

### Phosphorylation of MyBP-C and TnI is not altered in AE3-null hearts during pacing

**Figure 7:**
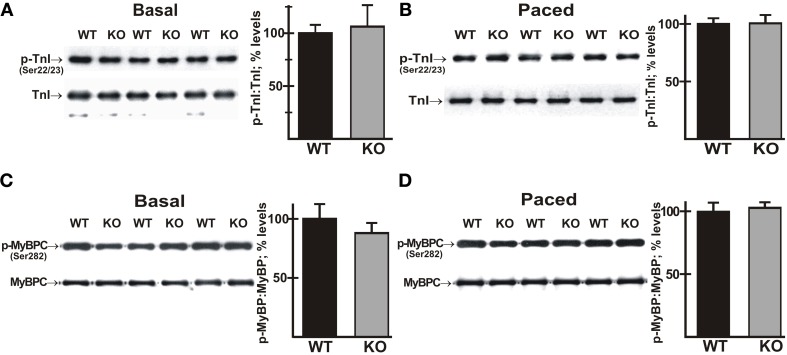
**Phosphorylation of cardiac troponin I (TnI) and myosin binding protein-C (MyBP-C) in wild-type and AE3-null hearts subjected to atrial pacing**. WT and AE3-null (KO) mice were anesthetized, instrumented, and subjected to pacing as described in Figure 2. Ventricular tissue was harvested under basal conditions in instrumented controls or during pacing at 550 bpm. Immunoblot and densitometric analyses of homogenates were carried out to determine levels of: **(A)** total TnI and phosphorylated TnI (Ser22/23) under basal conditions; **(B)** total TnI and phosphorylated TnI (Ser22/23) during pacing; **(C)** total MyBP-C and phosphorylated MyBP-C (Ser282) under basal conditions; and **(D)** total MyBP-C and phosphorylated MyBP-C (Ser282) during pacing at 550 bpm. Results show representative immunoblots, and percent relative levels (normalized to total TnI or MyBP-C levels) of phosphorylated TnI or MyBP-C. The two genotypes exhibited no differences in either total TnI or total MyBP-C. Values shown are means ± SE, *n* = 4 mice for each genotype and pacing condition.

Mutations in a number of myofibrillar proteins, including TnI and MyBP-C, have been identified in HCM (Frey et al., 2011; Marston et al., 2012), and phosphorylation of both TnI and MyBP-C (Tong et al., 2004; Bilchick et al., 2007; Zhang et al., 2011) have been shown to affect frequency-dependent responses. Immunoblot analyses of total cardiac homogenates revealed that the expression levels of both proteins and the relative phosphorylation levels of TnI (Ser22/23) and MyBP-C (Ser282) were not significantly altered in mutant hearts under either basal conditions (Figures 7A,C) or after pacing to 550 bpm (Figures 7B,D).

### Phosphorylation of Akt is increased in AE3-null hearts during pacing

Besides myofibrillar proteins, mutations in proteins involved in biomechanical stress sensing and responses have been implicated in both regulation of contractility (Bendig et al., 2006; Meder et al., 2011) and HCM (Frey et al., 2011). The reduction in positive FFR indicated that the contractile response to acute biomechanical stress was deficient in AE3-null hearts. Thus, it seemed possible that alterations in biomechanical stress sensing and/or responses might be involved in the susceptibility of AE3-null mice to heart failure in HCM.

AE3 has been localized to t-tubules and to the sarcolemma, where it appears to be associated with costameres (Alvarez et al., 2007a). Integrin-linked kinase (ILK), which interacts with β1-integrin, is a component of a sensor/signaling complex that is located at the costamere and plays a role in biomechanical stress sensing and signaling (Meder et al., 2011). Interestingly, the kidney AE1 Cl^−^/HCO^−^_3_ exchanger, a closely related member of the *Slc4a* family that is expressed in α-intercalated cells of the collecting duct, has been shown to interact with ILK (Keskanokwong et al., 2007). The IPP complex initiates mechanosensory signaling cascades in heart, and phosphorylation of Ser473 of Akt is a key signaling event in this pathway (Brancaccio et al., 2006; Srivastava and Yu, 2006). Activation of Akt can contribute to cardiac contractility (Condorelli et al., 2002), but its long-term activation can also lead to hypertrophy and heart failure (Chaanine and Hajjar, 2011; Sussman et al., 2011), suggesting that it might be involved in the AE3 phenotype.

**Figure 8:**
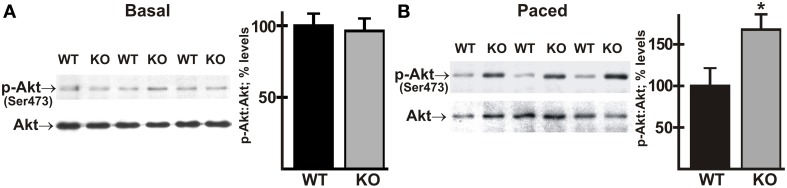
**Phosphorylation of Akt in wild-type and AE3-null hearts subjected to atrial pacing *in vivo***. WT and AE3-null (KO) mice were anesthetized, instrumented, and subjected to pacing as described in Figure 2. Ventricular tissue was harvested under basal conditions in instrumented controls or during pacing at 550 bpm. Immunoblot and densitometric analyses of homogenates were carried out to determine levels of: **(A)** total Akt and phosphorylated Akt (Ser473) under basal conditions; and **(B)** total Akt and phosphorylated Akt (Ser473) during pacing at 550 bpm. Results show representative immunoblot and percent relative levels (normalized to total Akt levels) of phosphorylated Akt. Values shown are means ± SE, *n* = at least 10 for each genotype and pacing condition. ^*^p < 0.05 vs. WT.

To determine whether activation of the Akt pathway was affected by the loss of AE3, phosphorylation of Akt on Ser473 was analyzed in ventricle tissue from WT and AE3-null mice under basal conditions and after *in vivo* pacing at 550 bpm. Under basal conditions there was no significant difference between WT and AE3-null hearts; however, Akt phosphorylation was significantly greater in hearts from AE3-null mice subjected to *in vivo* pacing when compared to hearts from corresponding WT controls (Figure 8; 168 ± 18% of WT levels). This suggests that biomechanical stress sensing and signaling via AKT responds more strongly in AE3-null hearts than in WT hearts.

### Phosphorylation of AMPK is decreased in AE3-null hearts during pacing

**Figure 9:**
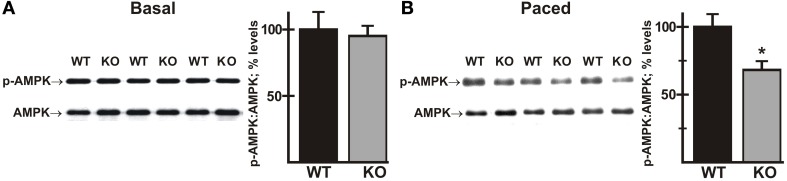
**Phosphorylation of AMPK in wild-type and AE3-null hearts subjected to atrial pacing *in vivo***. WT and AE3-null (KO) mice were anesthetized, instrumented, and subjected to pacing as described in Figure 2. Ventricular tissue was harvested under basal conditions in instrumented controls or during pacing at 550 bpm. Immunoblot and densitometric analyses of homogenates were carried out to determine levels of: **(A)** total AMPK and phosphorylated AMPK (Thr172) under basal conditions; and **(B)** total AMPK and phosphorylated AMPK (Thr172) during pacing at 550 bpm. Results show representative immunoblot and percent relative levels (normalized to total AMPK levels) of phosphorylated AMPK. Values shown are means ± SE, *n* = at least 8 for each genotype and pacing condition. ^*^p < 0.05 vs. WT.

There is evidence that activated Akt has a negative regulatory effect on AMP-activated protein kinase (AMPK) (Kovacic et al., 2003; Soltys et al., 2006). AMPK serves as a master regulator of cellular energy pathways (Zaha and Young, 2012), and mutations in the *PRKAG2* gene, encoding a regulatory subunit of AMPK, are known to cause HCM (Blair et al., 2001; Arad et al., 2002; Banerjee et al., 2010). This raises the possibility that perturbations of AMPK activity, secondary to enhanced Akt activation, might contribute to the heart failure phenotype in the AE3/TM180 HCM model (Al Moamen et al., 2011). To test this hypothesis we analyzed phosphorylation of AMPK on Thr172, which causes a sharp increase in enzyme activity (Zaha and Young, 2012). As observed for Akt phosphorylation, no differences in phosphorylation were observed under basal conditions (Figure 9A); however, AMPK phosphorylation was reduced in AE3-null hearts relative to WT controls when paced *in vivo* at 550 bpm (Figure 9B), suggesting that impairments of energy metabolism may also contribute to the AE3-null phenotype.

## Discussion

Previous studies of the AE3-null mouse on an otherwise wild-type background revealed no evidence of a deficit in cardiovascular performance (Prasad et al., 2008). However, when combined with the α-tropomyosin Glu180Gly HCM mutation, the loss of AE3 led to more rapid decompensation and heart failure (Al Moamen et al., 2011). Those findings indicated that ablation of AE3 makes the heart more susceptible to heart failure in HCM, suggesting that AE3-mediated Cl^−^/HCO^−^_3_ exchange plays an important role in cardiac function. In the current study, we analyzed the effects of AE3 ablation on regulatory mechanisms known to contribute to normal contractile function in heart, including some that are affected in HCM. The data suggest that loss of AE3-mediated Cl^−^/HCO^−^_3_ exchange perturbs the ability of the heart to respond effectively to acute biomechanical stress.

IVP analyses of basal contractility and β-adrenergic responses was carried out using mice on an inbred FVB/N background. Although similar studies were carried out previously using WT and AE3-null mice of the 129Svj and Black Swiss mixed background (Prasad et al., 2008), there is evidence that the genetic background can have pronounced effects on inotropic responses in mice (Kadambi et al., 1999). The reasons underlying these background-specific differences are unclear, but may involve variations in Ca^2+^-handling and the expression, localization, or activation of key signaling molecules. Nevertheless, cardiovascular performance under basal conditions and the chronotropic, inotropic, and lusitropic effects of β-adrenergic stimulation were largely normal in AE3-null mice of either background (Prasad et al., 2008; current study).

Frequency-dependent augmentation of contractility is a key intrinsic regulatory mechanism in heart, and in a healthy myocardium the relationship between increased heart rate and contractility is positive (Endoh, 2004). Although less robust than in larger mammals such as humans, FFR in mice seems to be qualitatively similar to FFR in larger mammals (Palakodeti et al., 1997). Because frequency-dependent responses are often impaired in heart failure (Rossman et al., 2004), cardiac performance was analyzed *in vivo* under basal conditions and after pacing to 550 bpm. Relative to WT mice, which developed a well-defined positive FFR, with increases in +dP/dt consistent with previous studies using atrial pacing in closed-chest mice (D'Angelo et al., 1997), the positive FFR was blunted in AE3-null mice. The effects in AE3-null mice were variable, with some AE3-null mice exhibiting a robust FFR. Also, as noted above, contractile dysfunction was not apparent during β-adrenergic stimulation. Nevertheless, the observed blunting of frequency-dependent inotropy provides the first direct evidence of contractile dysfunction due to the loss of AE3 alone.

The mean values for pacing-induced differences in −dP/dt, although not statistically significant, were reduced in AE3-null mice, suggesting the possibility of diastolic dysfunction. For example, FDAR is impaired in humans exhibiting heart failure with normal ejection fraction (Wachter et al., 2009). However, tau values and LVEDP, which are more reliable measures of relaxation and diastolic dysfunction (Weiss et al., 1976), were both significantly elevated in those patients, whereas in AE3-null mice these parameters were not significantly different from those in WT controls. Furthermore, phosphorylation of PLN and TnI, both of which affect relaxation (Wolska et al., 2002), were not changed in the pacing experiments. Thus, impairment of frequency-dependent effects in AE3-null mice appears to be limited to contractility.

Ca^2+^ is a major regulator of cardiac function, and altered expression of Ca^2+^-handling proteins has been correlated with impaired contractility (Periasamy et al., 1999; Pieske et al., 1999; Seidler et al., 2007). Although AE3-null hearts exhibited normal basal contractility and β-adrenergic responses, immunoblotting revealed increased expression of SERCA2 and NCX1, which together account for most of the Ca^2+^-clearance from the cytosol during diastole, and reduced expression of LTCC, which mediates Ca^2+^-influx on a beat-to-beat basis. Despite these changes, Ca^2+^-handling was unaltered in AE3-null myocytes, as indicated by no differences in peak Ca^2+^ or in the rate of decay of the Ca^2+^ transient in electrically stimulated myocytes *in vitro*. The observed changes in expression of Ca^2+^-handling proteins and the Ca^2+^ imaging experiments in isolated myocytes do not support the hypothesis that perturbations of Ca^2+^-handling are the primary cause for the reduction in rate-dependent inotropy. However, as discussed below, these data do not rule out the possibility that impairment of Ca^2+^ handling *in vivo* contributes to susceptibility to HCM or to the blunting of the positive FFR in AE3-null mice.

A positive FFR has been observed in isolated mouse myocytes, in a range of frequencies (up to 300 bpm) that are below those occurring *in vivo*, and appeared to be dependent on increased phosphorylation of PLN on Thr17 (Zhao et al., 2004), which increases SERCA2 activity. However, phosphorylation of Thr17 and Ser16 was similar in hearts of AE3-null and WT mice during pacing *in vivo*, indicating that reduced phosphorylation of PLN, which would limit SERCA2 activity, does not contribute to the differences in FFR between the two genotypes. Furthermore, a reduction in SERCA2 expression has been shown to cause blunting of the positive FFR in mice (Huke et al., 2003). Thus, the increased expression of SERCA2 observed in hearts of AE3-null mice would be expected to improve, rather than impair, contractility and the positive FFR. Similarly, the increase in NCX1 and NHE1 levels could contribute to increased Ca^2+^-loading via reverse mode activity of the Na^+^/Ca^2+^ exchanger, thereby leading to improved contractility. In fact, increased NCX1 expression has been associated with better preservation of both systolic (LaRocca et al., 2012) and diastolic (Hasenfuss et al., 1999) function in heart failure. The likely effects of decreased LTCC expression on frequency-dependent inotropy is difficult to predict. Either pharmacologic stimulation or inhibition of the LTCC in muscle strips of human failing heart caused an increase in the positive FFR, although basal contractility was reduced by inhibition of channel activity (Reuter et al., 1999). Overall, our results suggest that alterations in Ca^2+^-handling or expression of Ca^2+^-handling proteins resulting from the loss of AE3, at least as it relates to direct modulation of the contractile apparatus, are unlikely to contribute to the reduction in rate-dependent inotropy or to the more rapid decompensation and heart failure observed in the HCM model (Al Moamen et al., 2011).

NBCe1 was shown previously to be upregulated in the retina of AE3-null mice (Alvarez et al., 2007b), an effect opposite to the reduction in NBCe1 expression seen in AE3-null hearts. NBCe1 can mediate Na:HCO^−^_3_ cotransport with a stoichiometry of either 1:2 or 1:3 (Gross and Kurtz, 2002). In heart it has a ratio of 1:2 and mediates inward cotransport (Garciarena et al., 2013). However, in retina the Na:HCO^−^_3_ ratio is 1:3 (Newman and Astion, 1991), as in kidney, which would allow outward transport of Na^+^ and HCO^−^_3_. In fact, Alvarez et al. (2007b) suggested that both AE3 and NBCe1 in the end feet of Muller cells mediate outward transport of HCO^−^_3_. Thus, an increase in NBCe1 expression in the retina would be expected to provide some compensation for the loss of HCO^−^_3_ extrusion via AE3. In contrast, loss of AE3 in heart would eliminate a major HCO^−^_3_ extrusion activity, so a reduction in HCO^−^_3_-uptake via NBCe1 would be expected to provide some degree of compensation with respect to intracellular HCO^−^_3_. Thus, the reduction in NBCe1 expression may compensate in part for perturbations of intracellular pH (pH_*i*_) that occur in response to loss of AE3; however, it could also affect Na^+^-loading.

NBCe1 and NHE1 have been identified as major acid extrusion mechanisms in cardiac myocytes (Garciarena et al., 2013). Both transporters mediate Na^+^-uptake, which in turn can increase contractility by Ca^2+^-loading via reverse mode activity of the Na^+^/Ca^2+^ exchanger. With expression in both t-tubules and sarcolemma (Garciarena et al., 2013), and relatively high expression in heart (Prasad et al., 2013), NBCe1 may be capable of Na^+^-loading in a manner that could affect Ca^2+^-loading. It has been shown that an increase in the frequency of contraction of papillary muscles in CO_2_/HCO^−^_3_ buffered media leads to an increase in pH_*i*_ that can be blocked by a Na^+^/HCO^−^_3_ cotransport inhibitor (Camilión de Hurtado et al., 1996). The authors concluded that an electrogenic Na^+^/HCO^−^_3_ cotransporter, now known to be NBCe1, is strongly activated by depolarization during each action potential and that Na^+^/HCO^−^_3_ cotransport activity increased as heart rate increased. Thus, it is possible that pacing to higher heart rates *in vivo* leads to a transient increase in both pH_*i*_ and Na^+^. If this were the case, then AE3-mediated HCO^−^_3_ extrusion would tend to maintain a strong driving force for NBCe1 activity by reducing intracellular HCO^−^_3_ and, because it is expressed in t-tubules (Alvarez et al., 2007a), AE3 would replenish HCO^−^_3_ concentrations in the lumen of the t-tubule. Thus, it is possible that the loss of AE3 and the reduction in NBCe1 expression could lead to a reduction in Na^+^-loading at elevated heart rates, which could affect Ca^2+^-loading, contractility, and the FFR.

An additional effect of changes in AE3 and NBCe1 activity might be alterations in the activity of L-type Ca^2+^ channels, which would also affect Ca^2+^-loading. It has been shown that a reduction in pH_*i*_ increases L-type Ca^2+^ currents, whereas a decrease in extracellular pH (pH_*o*_) decreases Ca^2+^ currents (Saegusa et al., 2011). By extruding HCO^−^_3_ across the sarcolemma or t-tubular membranes, AE3 would be expected to reduce pH_*i*_ and increase pH_*o*_ in its immediate vicinity, which would be predicted to increase Ca^2+^ currents, whereas NBCe1 activity would be expected to have the opposite effect. Also, it has been suggested that NBCe1 activity, because of its anionic current, reduces the duration of the action potential (De Giusti et al., 2011). Thus, the loss of AE3 could lead to a reduction in L-type Ca^2+^ currents, with a consequent reduction in contractility. A reduction in NBCe1 activity, by reducing the magnitude of these pH perturbations and increasing action potential duration, might provide some compensation. The remarkably strong co-localization of NBCe1 and L-type Ca^2+^ channels (Garciarena et al., 2013) supports the possibility that NBCe1 regulates Ca^2+^ channel activity. Additional studies will be needed to assess these possibilities.

Along with Ca^2+^-handling, alterations in myofibrillar Ca^2+^-responsiveness are known to regulate contractility (Allen and Kentish, 1985; Kentish, 1986; Ruegg, 1987), and phosphorylation of myofibrillar proteins such as MyBP-C and TnI plays a key role in determining Ca^2+^-sensitivity, and therefore contractility (Kentish et al., 2001; Solaro, 2002; Barefield and Sadayappan, 2010). In addition, mutations in both TnI and MyBP-C are known causes of HCM (Frey et al., 2011; Marston et al., 2012). However, we observed no alterations in the expression or phosphorylation of MyBP-C or TnI, suggesting that changes involving these proteins do not contribute to the blunted FFR or to the increased susceptibility to HCM and decompensation in heart failure observed in AE3-null mice.

An observation made during Ca^2+^ imaging analysis of isolated myocytes may provide a clue to a deficiency caused by the loss of AE3. Isolated AE3-null myocytes stimulated at relatively low frequencies had a greater tendency than WT myocytes to round up. The basis for this structural change has not been investigated further, but rounding up of ventricular myocytes in culture is associated with breakdown of the cytoskeleton or dissociation of costameres from Z-lines of the myocyte (Imanaka-Yoshida et al., 1996; Weikert et al., 2003). The presence of AE3 at costameres (Alvarez et al., 2007a) is consistent with a role in cytoskeletal integrity, a feature that it may share with other anion exchangers of the *Slc4a* family. AE1, the Band 3 Cl^−^/HCO^−^_3_ exchanger, has a well-known role in maintaining cytoskeletal integrity of the erythrocyte (Peters et al., 1996; Southgate et al., 1996) and, in osteoclasts, AE2 activity regulates the reorganization of actin superstructures and disassembly of podosomes (Coury et al., 2013). Podosomes and costameres are similar in that they are integrin-containing structures that interact with both the cytoskeleton and extracellular matrix and serve as major centers of mechanosensing and signaling (Samarel, 2005; Schachtner et al., 2013). For AE2, it has been shown that its Cl^−^/HCO^−^_3_ exchange activity is necessary for normal podosome function; however, the N-terminal cytoplasmic domain that interacts with cytoskeletal components is not essential (Coury et al., 2013), suggesting that direct physical interactions with the cytoskeleton are not necessary.

The ability to sense and respond to changes in mechanical stress is a critical regulatory mechanism in heart (Hoshijima, 2006; Heineke and Molkentin, 2006). Costameres form structural links between Z-discs and the extracellular matrix and serve as integrative nodal points in the mechanosensory machinery (Samarel, 2005). The IPP complex, a sub-sarcolemmal multiprotein complex that includes integrins and ILK, is critical for mechanosensing at costameres (Brancaccio et al., 2006; Srivastava and Yu, 2006), and Akt has been identified as a key player in mechanosensory signaling via this complex (Meder et al., 2011). We therefore explored the possibility that Akt activity, as evidenced by phosphorylation of Akt on Ser473, was affected in mutant hearts. Although Akt phosphorylation was the same in mutant and WT hearts under basal conditions, p-Akt levels were significantly higher in AE3-null hearts when subjected to *in vivo* pacing, indicating that this mechanosensitive signaling pathway was activated to a greater degree in AE3-null hearts than in WT hearts. It should be noted that the increase in p-Akt levels during pacing would not be expected to contribute to the blunting of rate-dependent inotropy because increased Akt activity has been shown to enhance contractility (Condorelli et al., 2002; Cittadini et al., 2006; Sussman et al., 2011). Rather, it is likely that the enhanced Akt phosphorylation is a compensatory mechanism that is required to maintain a more normal FFR in AE3-null hearts.

Given its critical signaling function in heart, with effects on contractility, muscle mass, myocyte survival, and energy metabolism (Sussman et al., 2011), the elevation of Akt activity, if it were chronic, would be likely to have major consequences in mutant hearts (Chaanine and Hajjar, 2011). The average basal heart rate in the anesthetized mice used in this study (~400 bpm) is well below the ~550–600 bpm we have measured by telemetry in awake WT mice (Bradford et al., 2010); however, the electrically paced heart rate of 550 bpm, where activated Akt was observed, is within the normal range for awake mice and much higher heart rates can be achieved when mice are active. Thus, it is possible that Akt is chronically activated in awake AE3-null mice. The mildly increased expression of SERCA2 and NCX1 could be due to chronically activated Akt as transgenic overexpression of Akt caused a sharp increase in the levels of SERCA2 (Kim et al., 2003; Cittadini et al., 2006) and NCX1 (Formisano et al., 2008).

The mechanisms by which loss of AE3 might lead to increased biomechanical stress or affect sensing and responses to such stress are unclear. We suggested previously that in cardiac myocytes, AE3 may act in concert with a Na^+^-loading acid extruder, regulating sub-sarcolemmal [Na^+^] and thereby modulating [Ca^2+^] in localized microdomains (Prasad et al., 2008) rather than in the bulk cytosol where it would be expected to affect contractility directly. The similar localization of AE3 (Alvarez et al., 2007a) and NBCe1 (Garciarena et al., 2013) and the down-regulation of NBCe1 in AE3-null mice suggest that AE3 might exhibit some degree of functional coupling with NBCe1. Given the evidence that spatially compartmentalized Ca^2+^-signals regulate mechanical stress-responsive signaling (Judice et al., 2009) one can speculate that the loss of AE3 might impact the role of Ca^2+^ in responses to mechanical stress. An alternative possibility is that loss of AE3 may impair responses to biomechanical stress by affecting subsarcolemmal or cytosolic pH_*i*_, HCO^−^_3_, or Cl^−^concentrations. For example, F-actin rearrangements that are dependent on cofilin 2 and interactions with muscle LIM protein (MLP or *Csrp3*) are strongly affected by pH_*i*_ (Papalouka et al., 2009), which would likely be more alkaline in subsarcolemmal domains following loss of AE3. HCO^−^_3_ has been shown to cause a sharp increase in contractility in isolated hearts, with no increase in systolic Ca^2+^, indicating a critical role for HCO^−^_3_ homeostasis (Fülöp et al., 2003). Regarding possible effects due to alterations of cellular Cl^−^ homeostasis, when the AE3 knockout was crossed with an NKCC1 Na^+^-K^+^-2Cl^−^ cotransporter knockout, the double mutant exhibited impaired contractility; however, it appeared likely that this was due to impaired Ca^2+^ loading as NCX1-mediated Ca^2+^ extrusion was increased (Prasad et al., 2008). Additional studies will be needed to address the molecular mechanisms by which loss of AE3 affects biomechanical stress specifically and cardiac function in general.

The data clearly show that Akt signaling is enhanced in AE3-null hearts during pacing. This would be expected to improve the heart's response to biomechanical stress; however, if Akt were chronically activated, as seems likely given the heart rates that occur naturally in awake mice, it could also have detrimental effects (Chaanine and Hajjar, 2011; Sussman et al., 2011). One such effect is impaired AMPK signaling (Kovacic et al., 2003; Soltys et al., 2006), which has been linked to genetic HCM (Blair et al., 2001; Arad et al., 2002; Banerjee et al., 2010). Phosphorylation of AMPK on Thr172 was the same in AE3-null and WT hearts of anesthetized mice under basal conditions, but when paced to 550 bpm, phosphorylation in AE3-null hearts was reduced, consistent with the possibility that enhanced AKT signaling causes a reduction in AMPK signaling. Reduced AMPK signaling would be expected to have a negative impact on metabolism (Dyck and Lopaschuk, 2006; Beauloye et al., 2011; Zaha and Young, 2012), which in turn could be part of the mechanism by which AE3-deficiency leads to more rapid decompensation in heart failure (Al Moamen et al., 2011).

In summary, our results show that the loss of AE3-mediated Cl^−^/HCO^−^_3_ exchange leads to an impaired FFR, which is a clear physiological correlate of the predisposition to heart failure in the TM180 model of HCM (Al Moamen et al., 2011). The results of immunoblot analyses of proteins involved in Ca^2+^ transport and Ca^2+^ imaging of isolated myocytes argue against the hypothesis that primary perturbations of Ca^2+^ handling are responsible for the physiological deficit, although it is possible that modulatory effects of AE3 and NBCe1 activities on Ca^2+^ channels could perturb Ca^2+^ handling at the high frequencies occurring *in vivo*. There were no differences in the levels of total or phosphorylated MyBP-C and TnI, making it unlikely that altered activities or Ca^2+^ sensitivity of these myofibrillar proteins contribute to the deficit. The most striking change was an increase in phosphorylation of Akt and a decrease in phosphorylation of AMPK during pacing at elevated heart rates. AE3 has been localized to both sarcolemmal and intracellular membranes, and on the sarcolemma it appears to be concentrated at costameres (Alvarez et al., 2007a). Because Akt is known to participate in biomechanical stress signaling originating at costameres (Brancaccio et al., 2006; Srivastava and Yu, 2006), its increased activation suggests that hearts of AE3-null mice are more sensitive to acute biomechanical stress than hearts of WT mice when subjected to *in vivo* pacing. The mechanisms by which loss of AE3 leads to a deficient response to biomechanical stress and the downstream events that lead to decompensation in heart failure, possibly including impaired AMPK activation, will be investigated in future studies.

## Author contributions

The experiments were conceived by Vikram Prasad, Gary E. Shull, and John N. Lorenz; the biochemical analyses were performed by Vikram Prasad; the analyses of cardiovascular function were performed by Valerie M. Lasko, Michelle L. Nieman, and John N. Lorenz; isolation of cardiomyocytes and Ca^2+^ imaging experiments were performed by Nabeel J. Al Moamen; data were analyzed by Vikram Prasad, Gary E. Shull, John N. Lorenz, and Nabeel J. Al Moamen; and the manuscript was written by Vikram Prasad and Gary E. Shull.

### Conflict of interest statement

The authors declare that the research was conducted in the absence of any commercial or financial relationships that could be construed as a potential conflict of interest.
